# Biotransporting Biocatalytic Reactors toward Therapeutic Nanofactories

**DOI:** 10.1002/advs.201800801

**Published:** 2018-09-19

**Authors:** Tomoki Nishimura, Kazunari Akiyoshi

**Affiliations:** ^1^ Department of Polymer Chemistry Graduate School of Engineering Kyoto University Katsura Nishikyo‐ku Kyoto 615‐8510 Japan; ^2^ ERATO Bio‐Nanotransporter Project Japan Science and Technology Agency (JST) Kyoto University Katsura Nishikyo‐ku Kyoto 615‐8530 Japan

**Keywords:** biocatalytic reactors, molecular permeable compartments, therapeutic nanofactories

## Abstract

Drug‐delivery systems (DDSs), in which drug encapsulation in nanoparticles enables targeted delivery of therapeutic agents and their release at specific disease sites, are important because they improve drug efficacy and help to decrease side effects. Although significant progress has been made in the development of DDSs for the treatment of a wide range of diseases, new approaches that increase the scope and effectiveness of such systems are still needed. Concepts such as nanoreactors and nanofactories are therefore attracting much attention. Nanoreactors, which basically consist of vesicle‐encapsulated enzymes, provide prodrug conversion to therapeutic agents rather than simple drug delivery. Nanofactories are an extension of this concept and combine the features of nanoreactors and delivery carriers. Here, the required features of nanofactories are discussed and an overview of current strategies for the design and fabrication of different types of nanoreactors, i.e., systems based on lipid or polymer vesicles, capsules, mesoporous silica, viral capsids, and hydrogels, and their respective advantages and shortcomings, is provided. In vivo applications of biocatalytic reactors in the treatment of cancer, glaucoma, neuropathic pain, and alcohol intoxication are also discussed. Finally, the prospects for further progress in this important and promising field are outlined.

## 1 Introduction

The development of drug‐delivery systems (DDSs) has attracted significant attention over the last 40 years. This has been driven by the importance of improving the clinical performances of various therapeutics and the need to develop novel diagnostic sensing and imaging tools. DDSs rely greatly on nanotechnology. For example, active therapeutic drugs are encapsulated in nanoparticles (e.g., micelles, vesicles, solid‐sphere particles, nanogels, and viruses), and delivered to, and are released at, desired places in living bodies. This enables enhancement of therapeutic effects and a reduction in side effects. Such nanotechnology‐based DDSs (i.e., nanomedicines) have been investigated for the treatment of many diseases. In cancer therapy, for instance, several nanomedicines such as Doxil, Abraxane, and Onivyde have been approved for clinical use and have significantly improved patient survival.^1^, ^2^ Other novel nanomedicines have shown superior anticancer activity in preclinical studies.^3^ Although these conventional nanomedicines have valuable therapeutic effects, their effects are limited to extending patient survival, and patients still suffer from side effects. There is therefore a growing need for different approaches to the development of novel nanomedicines.

In addition to conventional DDSs, the concept of using enzymatic reactions to convert a prodrug to a therapeutic drug (known as enzymatic prodrug therapy, EPT) has been used for DDS development since the mid‐1980s. Nishiyama et al. reported the use of cytosine deaminase (CD) encapsulated in a dialysis bag as an implantable device for local enzymatic prodrug conversion.^4^ They reported that local implantation of capsules containing CD and subsequent addition of 5‐fluorocytosine (5‐FU) effectively converted 5‐FU to 5‐fluorouracil, which led to inhibition of tumor growth.

This work showed that EPT is a powerful therapeutic tool and various types of EPTs have been developed to improve therapeutic efficiency. For example, Bagshawe reported an enzyme conjugated antibody that can deliver the enzyme to a specific disease site where the enzyme can convert a prodrug into an active drug.^5^ The released drug can be diffused into cells (e.g., cancer cells), leading to cell death. This method is known as antibody‐directed enzyme prodrug therapy (ADEPT). Because of these superior therapeutic effects, several clinical trials using ADEPT have been performed.^6^ Drug‐conjugated polymers have also been used to construct enzyme prodrug activation systems for polymer‐directed enzyme prodrug therapy (PDEPT).^7^, ^8^ This system consists of a drug immobilized on a polymer via a cleavable linker for selective inter‐/intracellular enzymatic reactions by specific enzymes at disease sites. In 2005, Gelder and co‐workers reported another EPT‐based concept, i.e., therapeutic nanoreactors.^9^, ^10^ These consist of a biocatalyst (enzyme) encapsulated in polymer vesicles equipped with a channel protein and are designed to transform prodrugs into therapeutic drugs by an encapsulated enzyme. EPT has great potential for the treatment of a variety of diseases, and many excellent reviews that cover the history of, and recent progress in, EPT are available.^11^, ^12^, ^13^

In 2007, a working group including chemists, engineers, and medical researchers extended the concept of such therapeutic reactors to more elaborate biocatalytic reactors, namely nanofactories.^14^ A nanofactory is a compartment with the characteristics of both a biocatalytic reactor and a delivery carrier; it performs specific functions at disease sites, in either intra‐ or intercellular environments. The difference between an EPT system and a nanofactory is that a nanofactory is designed to act mainly in intracellular environments rather than extracellular environments. More specifically, nanofactories can transport enzymes to intracellular environments, at which toxic materials or prodrugs are selectively permeated inside the nanofactories and transformed into harmless compounds or native drugs. Such in situ production of therapeutic drugs and detoxification has distinct advantages over conventional DDSs, namely a reduction in unwanted side effects and enhanced therapeutic efficacy.

**Figure 1:**
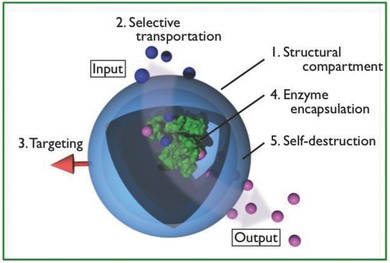
Requirements for therapeutic nanofactories.

The working group reported that five requirements (**Figure** **1**) need to be fulfilled by such nanofactories (i.e., therapeutic biocatalytic reactors): 1) the presence of a structural compartment; 2) transportation of specific molecules to and from the outer environment; 3) accumulation at the target site; 4) enzyme encapsulation, and 5) self‐destruction in response to an external trigger. The construction of such nanofactories is therefore a great challenge. In recent years, however, compartments that fulfill several of the above requirements have been developed and have potential for nanofactory production.

This review aims to describe various strategies for the fabrication of biocatalytic reactors. The major merits of each strategy are highlighted and the remaining drawbacks are also discussed. Such biocatalytic reactors have also been used for the construction of artificial cells or organelles, and there are a large number of literature reports and excellent reviews on this topic,^15^, ^16^, ^17^, ^18^, ^19^, ^20^ but this is beyond the scope of this review. Finally, we will highlight some of the challenges in the use of biocatalytic reactors for in vivo medical applications.

## 2 Design Strategies and Functions of Biocatalytic Reactors

**Figure 2:**
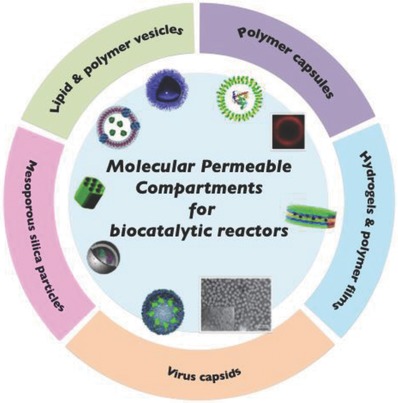
Schematic diagram of five different compartments in scaffold of biocatalytic reactor. Lipid & polymer vesicles, left: Reproduced with permission.^62^ Copyright 2016, Wiley‐VCH, right: Reproduced with permission.^72^ Copyright 2017, Wiley‐VCH. Polymer capsules, left: Reproduced with permission.^84^ Copyright 2012, American Chemical Society, right: Reproduced with permission.^88^ Copyright 2010, Wiley‐VCH. Mesoporous silica particles, top: Reproduced with permission.^131^ Copyright 2013, American Chemical Society, bottom: Reproduced with permission.^134^ Copyright 2014, Wiley‐VCH. Virus capsids, left: Reproduced with permission.^137^ Copyright 2007, Springer Nature, right: Reproduced with permission.^141^ Copyright 2014, Royal Society of Chemistry. Hydrogels & polymer film, Reproduced with permission.^149^ Copyright 2014, The Royal Society of Chemistry.

Enzymes are biocatalysts that can accelerate a wide variety of reactions such as decomposition of harmful compounds. Consequently, enzymes can be regarded as medicines.^21^ However, externally introduced enzymes are easily denatured in living bodies (e.g., in the blood stream and inside cells), and are recognized by the immune system, leading to exclusion. They therefore need to be protected from the external environment by placing them in structural compartments. The cells and organelles in living systems have biological membranes, which are composed of lipid bilayers. Bilayer membranes separate the inner aqueous phase from the outer environment by compartmentalization. This provides integration of functional molecules such as enzymes and a specific reaction space. The compartmentalization of enzymes is a result of natural selection and therefore provides a rational approach to the performance of effective enzymatic reactions. In addition to enzyme entrapment, compartments acting as therapeutic biocatalytic nanoreactors must fulfill the five requirements listed in the introduction. Much effort has been made to develop compartments that can act as biocatalytic reactors.^22^ In the following section, we provide an overview of strategies for designing biocatalytic reactors based on various scaffolds (e.g., lipid and polymer vesicles, capsules, mesoporous silica, viral capsids, and hydrogels) and their biocatalytic activities in aqueous solution and intracellular environments (**Figure** **2**).

### 2.1 Lipid Vesicles

Lipid vesicles, or liposomes, which are constructed by self‐assembly of certain phospholipids, have one or more closed compartment(s). The compartments retain various molecules, including enzymes, without leakage, and have moderate stability. They are therefore suitable structural components for biocatalytic reactors. Although lipid vesicles are good candidates for reactors, as mentioned before, they have the drawback of low molecular permeability. Because of their low permeability, externally added substrates hardly permeate into lipid vesicle interiors, therefore the enzyme reaction cannot occur inside the vesicles.^23^ In the following section, we will discuss strategies for enhancing the molecular permeability of lipid vesicles.

#### 2.1.1 Temperature‐Responsive Vesicles

Lipid bilayers show phase‐transition temperatures (*T*_m_). When a liposome solution is heated, the bilayer undergoes a transition from a gel phase to a ripple phase and then a liquid‐crystal phase. During phase separation, the mobility and fluidity of the lipid molecules in the membrane can be altered. The bilayer membrane in the liquid‐crystal phase is more permeable than that in the gel phase. Researchers have used this property in liposome‐based biocatalyst reactors. For example, Kaszuba and Jones prepared glucose oxidase (GOD)‐loaded dimyristoylphosphatidylcholine (DMPC; *T*_m_ = 23.6 °C) and measured the GOD activities at various temperatures.^24^ At 10 °C, the GOD activity was negligible, and the activity was the highest at 20 °C. This is consistent with the *T*_m_ values of DMPC.

**Figure 3:**
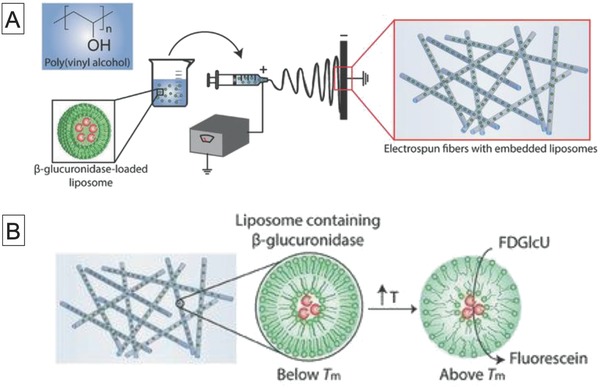
Schematic diagrams of A) preparation of electrospun poly(vinyl alcohol) (PVA) fibers with embedded β‐glucuronidase‐loaded liposomes and B) enzymatic conversion of fluorescein diglucuronide to fluorescein by PVA fibers containing β‐glucuronidase‐loaded liposomes at 37 °C, which is above the *T*_m_ of 1,2‐dioleoyl‐glycero‐3‐phosphocholine (DOPC) lipid. Reproduced with permission.^25^ Copyright 2017, Wiley‐VCH.

More recently, Stevens and co‐workers developed electrospun fibers with embedded liposome‐encapsulated enzymes for biocatalytic matrices, as shown in **Figure** **3**.^25^ They used DMPC and 1,2‐dipalmitoyl‐glycero‐3‐phosphocholine (DPPC) phospholipids for the preparation of liposomes and encapsulated β‐glucuronidase (β‐Glu) in the vesicles, followed by processing into nonwoven fibers by electrospinning. The β‐Glu activities of the liposomes embedded in the fibers were evaluated on the basis of conversion of fluorescein di‐β‐glucuronide. At 37 °C, the fluorescence signal derived from fluorescein was four times higher than that at 20 °C because of enhanced permeability of the substrate above the phase‐transition temperature of DMPC. They further demonstrated the therapeutic use of this biocatalytic matrix by producing an antiproliferative drug (SN‐38) in the presence of HeLa cells.

#### 2.1.2 Reconstituted Membrane Protein Vesicles

Cells and organelles take up nutrients via membrane channel proteins. Inspired by these natural systems, researchers have used membrane channel proteins to increase the molecular permeability of lipid vesicles. Meier and co‐workers reported channel membrane proteins (porin, OmpF) embedded in polymer‐stabilized phospholipid liposomes. The embedded OmpF acted as a channel, and allowed permeation of low‐molecular‐weight compounds (less than 400 g mol^−1^). This enabled liposome‐encapsulated β‐lactamase to act as a nanoreactor for hydrolysis of the antibiotic ampicillin inside the liposome.^26^ Nakao and co‐workers developed tandem biocatalytic reactors using OmpF incorporated into liposomes.^27^ The liposomes contained GOD and catalase (CAT) in the inner phase, and glucose was fed through the OmpF protein from the outer phase. The fed glucose was successfully transformed to gluconic acid and H_2_O_2_ by GOD, followed by decomposition of H_2_O_2_ to water and oxygen by CAT.

#### 2.1.3 Transient Hole Formation by Detergents

The methods described in Sections 2.1.1 and 2.1.2 (temperature and reconstitution of membrane proteins) are the main strategies for enhancing permeability, but another technique has been developed. Schmidt and co‐workers found that the addition of sodium cholate to liposomes induced the formation of transient holes in the bilayer membrane. The polysaccharide inulin, with an average molecular weight of 7 × 10^4^ g mol^−1^, can be loaded into the liposomes via the transient holes.^28^, ^29^, ^30^ Oberholzer et al. used this method for saccharide chain elongation of glycogen by the reaction of phosphorylase and its substrate glucose monophosphate,^31^ and polyadenine formation by the reaction of polynucleotide phosphorylase and adenosine 5′‐diphoshphate inside liposomes.^32^ Sodium cholate and triton X‐100^33^ both increased the phospholipid bilayer membrane permeability.

#### 2.1.4 Organic–Inorganic Hybrid Vesicles

**Figure 4:**
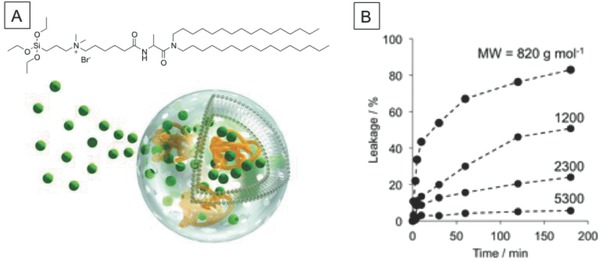
A) Chemical structure of organoalkoxysilane lipid and schematic diagram of semipermeable organic–inorganic hybrid vesicle and B) molecular weight–dependent release of FITC–PEGs with different molecular weights from organic–inorganic hybrid vesicles. Reproduced with permission.^34^ Copyright 2013, The Royal Society of Chemistry.

Another approach to the fabrication of permeable lipid vesicles is to use organic–inorganic hybrid vesicles, as reported by Yasuhara et al.^34^ The lipid vesicles consisted of an organoalkoxysilane lipid, i.e., *N*,*N*‐dihexadecyl‐*N‐α*‐{6‐[(3‐triethoxysilylpropyl)dimethylammonio]hexanoyl}alaninamide} bromide, and were stabilized by formation of a crosslinked siloxane (Si–O–Si) network on the membrane surface (**Figure** **4**). The resulting vesicles acted as molecular sieves and allowed permeation of hydrophilic molecules of molecular weights less than 1.5 kg mol^−1^. Because of this semipermeability, benzoyl‐l‐tyrosine‐*p*‐nitroanilide passed through the membrane and α‐chymotripsin encapsulated inside the vesicles successfully hydrolyzed the substrate. The proposed permeability mechanism is associated with the formation of domains with an oligosiloxane surface. This leads to packing defects at the domain boundary, and these facilitate permeation. This semipermeability and structural stability endowed by crosslinking would be beneficial in therapeutic biocatalytic nanoreactors.

### 2.2 Polymer Vesicles

Although liposomal compartments with enhanced molecular permeability can be used in biocatalytic nanoreactors, their stabilities in physiological environments are limited. Enhancement of the membrane stability can therefore be of considerable benefit in biocatalytic reactors in vivo. In 1995, Eisenberg and co‐workers discovered amphiphilic block copolymers, which are structural analogs of phospholipids, and could form spherical vesicles.^35^, ^36^ Shortly afterward, Discher et al. coined the term polymersome for this type of vesicle.^37^ The bilayers of polymer vesicles are much thicker than those of phospholipid membranes, and have the advantages of superior stability and toughness.^38^ The membrane stability increases with increasing thickness (i.e., molecular weight of the polymer), but the permeability decreases considerably. Accordingly, substantial efforts have been made to develop functional polymer vesicles with improved molecular permeability. The chemical properties can be engineered by changing the molecular weight and chemical structure of the block segment, therefore a wide variety of biocatalytic nanoreactors based on polymer vesicles have been reported. In the next section, some key examples are described.

#### 2.2.1 Stimuli‐Responsive Polymer Vesicles

A wide variety of stimuli‐responsive polymer vesicles that can undergo physical and chemical changes such as swelling, disassembly, and bond cleavage in response to a specific stimulus (e.g., pH, temperature, and light) have been reported.^39^ These vesicles are mainly designed for the controlled release of a loaded drug at a desired place in living cells or specific tissues. Some stimuli‐responsive vesicles are designed to enhance the membrane permeability in response to external stimuli. In the following section, we will describe the design of polymer vesicles that can alter the molecular permeability by responding to external stimuli, and their functions as biocatalytic reactors.

*pH‐Responsive Polymer Vesicles*: Significant acidification of tissues occurs in response to irregular physiological states such as inflammation and cancer. Acidified cellular components (lysosomes) are also present in living cells. Consequently, polymer vesicles with permeability that can be controlled by pH changes can provide site‐specific biocatalytic reactors. The first example of polymer vesicles equipped with pH‐responsive transmembrane channels was reported by Chiu et al. in 2008.^40^ The vesicles were produced by self‐assembly of copolymers of acrylic acid (AAc) and 1,2‐distearoylglycerol acrylate (DSA). The authors suggested that the DSA part of the copolymer was self‐assembled into a bilayer and AAc formed domains in the membrane. These AAc domains acted as pH‐responsive transmembrane channels. Calcein (a water‐soluble fluorophore) passed through the membrane at pH 8.0 but permeation by calcein was prohibited at pH 5.0. At pH 8, the vesicles were permeable not only to calcein but also to high‐molecular‐weight compounds such as hemoglobin. AAc ionization increased with increasing pH of the vesicle suspension, and this facilitated disruption of hydrogen bonds. This enhanced the molecular permeability when the pH increased. However, the sizes of these vesicles are too large for systemic injection and reduction to the nanometer scale is needed.

Voit and co‐workers reported another approach to modulate the permeability of polymer vesicle membranes, which involves the use of pH‐sensitive photo‐crosslinkable copolymers.^41^, ^42^ Their polymers consisted of biocompatible poly(ethylene glycol) (PEG) as a hydrophilic segment, pH‐sensitive diethylaminoethyl methacrylate (DEAEMA), and a photo‐crosslinking unit, i.e., 3,4‐dimethylmaleic imidobutyl methacrylate (DMIBM). Polymer vesicles of average size 120 nm were obtained by a pH switching method. Photoirradiation of the vesicle solution resulted in crosslinking of the DMIBM units in the membrane, which led to the formation of crosslinked polymer vesicles. These polymer vesicles showed pH‐dependent swelling/deswelling behavior because of the pH responsiveness of the poly(DEAEMA) (PDEAEMA) moiety. At high pH (pH 10), the DEAEMA chains are unprotonated and hydrophobic, leading to association, whereas they are protonated and hydrated at pH 4. Reversible swelling can be achieved by these pH changes. The vesicles were permeable in the acidic state and acted as bioreactors for the oxidation of guaiacol by myoglobin (Mb). A more interesting property of this vesicle is that the permeability is enhanced by shear flow, probably because of pore formation. This unique feature could enable the use of therapeutic nanofactories in blood vessels.

The same research group also reported another pH‐responsive polymer vesicle, which was based on PEG‐*b*‐poly(DEAEMA‐stat‐2‐hydroxy‐4‐(methacryloyloxy)benzophenone.^43^, ^44^ Du and co‐workers also developed photo‐crosslinked polymer vesicles that can transport bio‐macromolecules (enzymes and small interfering RNA (siRNA)) inside the vesicle lumen on the basis of pH changes.^45^

**Figure 5:**
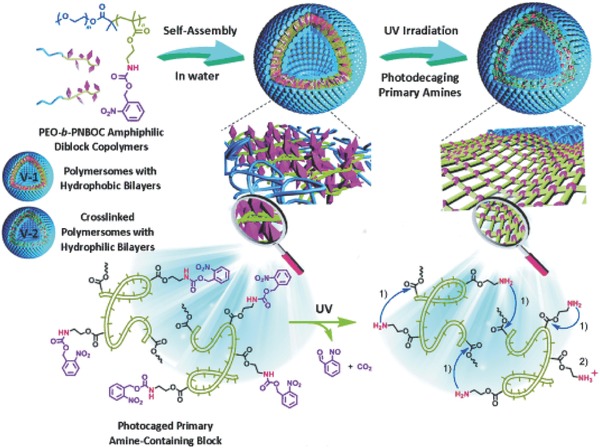
Chemical structure of photoresponsive amphiphilic block copolymer and schematic diagram of vesicles, showing phototriggered traceless crosslinking and vesicle membrane permeabilization. Reproduced with permission.^46^ Copyright 2014, Wiley‐VCH.

*Light‐Responsive Polymer Vesicles*: The pH‐responsive systems described above involve a two‐step procedure for fabrication of permeable vesicles. The first step is crosslinking for stability enhancement, and the second step is permeability control by changing the pH. Such systems are unsuitable if both stability and permeability are needed because these characteristics are incompatible. Consequently, another approach is needed. Liu and co‐workers are at the forefront of research on this issue. They have developed a new strategy, which involves light‐induced traceless crosslinking of polymer vesicles, as shown in **Figure** **5**.^46^ This strategy is based on amphiphilic block copolymers with a hydrophobic unit containing photolabile carbamate‐caged primary amine moieties (i.e., 2‐nitrobenzyloxycarbonylaminoethyl methacrylate). The polymers are self‐assembled into vesicles and on irradiation with UV light, the nitrobenzyl moieties are deprotected and primary amines are released. This results in the formation of inter‐ and intramolecular amide bonds. This leads to membrane crosslinking, accompanied by hydrophobic to hydrophilic transitions of the membrane. This feature enables the fabrication of permeable vesicles. For example, doxorubicin, which is a hydrophilic molecule, can be encapsulated inside the vesicle and released by UV irradiation. The release rate increases with increasing UV irradiation time, therefore the crosslinking density can be controlled by the irradiation time. The authors used this system to fabricate biocatalytic nanoreactors with switch on/off properties. They chose alkaline phosphatase (ALP) as a model enzyme for encapsulation in the vesicles. On UV irradiation, the vesicles were converted to permeable vesicles, and the nonfluorescent ALP substrate was able to pass through the membrane. The resulting substrate was hydrolyzed by ALP inside the vesicles to yield a fluorescent product.

The vesicle permeability can be altered by light irradiation, but the process is irreversible and a by‐product, which could be cytotoxic, is produced. Shortly after this report, they improved the system and developed polymer vesicles with molecular permeability that can be reversibly switched.^47^ In this system, photoswitchable spyropyran was incorporated into the hydrophobic segment of the amphiphilic polymer. On irradiation with light, the spyropyran was transformed into merocyanine, and the polymer membrane became disordered. This facilitated membrane permeability. When the vesicle solution was exposed to visible light, merocyanine was converted to the original spyropyran, and the membrane became impermeable.

Light has several advantages over other stimuli: mild activation, precise remote spatiotemporal control, and ease of use. However, visible or UV light does not penetrate deep into tissues because of light absorption and scattering. This hampers potential use of these light sources in further in vitro and in vivo biomedical applications. Although the use of near‐infrared (NIR) light in the range 700–1000 nm, which is the optical tissue penetration window, is one solution to this issue, NIR light–responsive systems have not yet been reported.

*Chemical‐Responsive Polymer Vesicles*: Another approach to functionalized permeability is to make use of the presence of specific molecules in pathological situations. van Hest and co‐workers reported the first example of polymer vesicle permeability induced by chemical stimuli.^48^ They formed vesicles by coassembly of PEG‐*b*‐poly(styrene) (PS) and PEG‐*b*‐poly(styrene boronic acid) (PSBA), which is a sugar‐responsive polymer. The vesicles have holes in their membranes when fructose (100 × 10^−3^ m) was present. The PSBA units bind to fructose, which increases the water solubility of PSBA. This results in disassembly of PEG–PSBA from the vesicles, but PEG–PS retains its structural integrity. This results in formation of vesicles with holes. As expected, the holes acted as transporting channels for small water‐soluble molecules such as carboxyfluorescein. The authors reported that *Candida antrarctica* lipase B (CALB) encapsulated in a polymersome nanoreactor catalyzed the hydrolytic reaction of 6,8‐difluoro‐4‐methylumbelliferyl octanoate (DiFMU octanoate).

**Figure 6:**
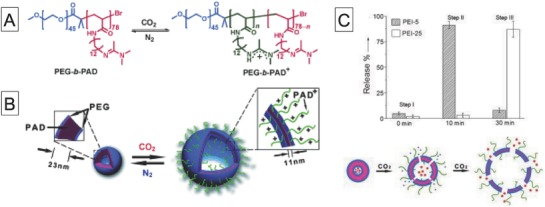
A) Chemical structure of CO_2_‐responsive amphiphilic block copolymer. B) Schematic diagram of self‐assembly of copolymer into polymer vesicles and reversible CO_2_‐controlled breathing behavior and C) amounts of released cargo after 10 min or 2 h of CO_2_ bubbling and schematic diagram of polymer vesicles that can permeate differently sized cargos depending on CO_2_ levels. Reproduced with permission.^49^ Copyright 2013, Wiley‐VCH.

Yuan and co‐workers developed another type of stimulus‐responsive polymer vesicle, with permeability that can be tuned by using CO_2_‐responsive polymers (**Figure** **6**).^49^ The vesicles were constructed by self‐assembly of PEG‐*b*‐poly[(*N*‐amidino)dodecyl acrylamine] (PAD). On exposure to CO_2_, the membrane thickness increased. This change is ascribed to a change in the protonation degree of the PAD unit, which accompanies hydration of the hydrophobic layer. During CO_2_ exposure, the membrane swelled, allowing permeation of hydrophilic molecules such as polyethyleneimine (PEI). More interestingly, molecular size–dependent permeability can be achieved by changing the CO_2_ exposure time; exposure for 10 min enabled permeation of PEI of molecular weight 5 kg mol^−1^, whereas PEIs of molecular weights 5 and 25 kg mol^−1^ both passed though the membrane after 30 min exposure. This property was used to elegantly show that on exposure to CO_2_, vesicles with entrapped Mb were permeable to O_2_ and glutathione, leading to the formation of O_2_‐carrying Mb inside the vesicles.

Although functionalization of the permeability of these systems is simple and they can be used as biocatalytic nanoreactors, a high concentration of sugar is needed because of low affinities between boronic acids and sugar molecules.^50^ Such high concentrations of sugar in living bodies are rarely observed. In addition, the CO_2_‐responsive systems involved direct CO_2_ bubbling, which is of little relevance in biological environments. Another approach to the design of polymer systems that respond to biologically relevant chemical species at the concentrations present in biological systems is therefore needed. Liu and co‐workers recently reported polymer vesicles that can be crosslinked and permeabilized by biologically relevant levels of H_2_O_2_, on the basis of their previous work.^51^ They prepared a PEG‐based amphiphilic block copolymer containing arylboronate ester–capped self‐immolative carbamate‐caged primary amine moieties. In the presence of H_2_O_2_, the arylboronate moieties were removed, followed by self‐immolative decaging reactions and generation of primary amine moieties. The resulting amine moieties reacted inter‐ and intramolecularly with ester groups to form amide bonds. This reaction induced vesicle crosslinking, as in their previous report. To test the applicability of this system inside living cells, mitochondria, which are organelles that generate reactive oxygen species (ROS), targeted H_2_O_2_‐reactive polymer vesicles were prepared. The vesicles were successfully accumulated in mitochondria, followed by transformation into permeable vesicles, as confirmed by release of the nucleus‐staining small molecule 4′,6‐diamidino‐2‐phenylindole (DAPI) dye. They also used this property for sustained drug release, magnetic resonance imaging, and fluorogenic nanoreactors. This system is especially notable for its ability to transform permeable vesicles even in crowded environments (i.e., organelles). It is difficult to control the size of the polymer vesicle and to reduce it to around 100 nm. Polymer vesicles of average diameter ≈500 nm were internalized into the cells; nanoparticles of similar sizes would immediately be entrapped by the reticuloendothelial system (RES) in vivo, so this system may not be suitable for use in systemic injections.

In addition to immobilizing stimuli‐responsive units on polymer vesicles, there is another interesting approach to developing permeable vesicles. This involves incorporation of a hydrophilic molecule into a hydrophobic segment of an amphiphilic polymer.^52^ Bruns and co‐workers showed that poly(2‐methyloxazoline)‐*b*‐poly(dimethylsiloxane)‐*b*‐(2‐methyloxazoline) with terminal acrylate groups and PEG‐*b*‐poly(butadiene)‐based polymer vesicles photoreacted with 2‐hydroxy‐4′,2‐methylpropiophenone (PPOH) to give polymer vesicles that allow low‐molecular‐weight molecules to penetrate the membrane from the external phase. This enables vesicles with encapsulated horse radish peroxidase (HRP) to oxidize a series of HRP substrates inside the vesicles. This study clearly proves that the photoreaction of PPOH with polymer vesicles is a simple method for inducing permeability, although the specific permeability mechanism is not clear. Such information will be useful in permeability control and will make this method more versatile.

*Temperature‐Responsive Polymer Vesicles*: Temperature is another stimulus that can be used to control membrane permeability. For this purpose, the well‐known temperature‐responsive polymer poly(*N*‐isopropylacrylamide) (PNIPAM) is often introduced into polymer systems to produce temperature‐responsive assemblies. PNIPAM solutions can switch from hydrophilic to hydrophobic at temperatures around the lower critical solution temperature (LCST). Ding and co‐workers used this property to develop temperature‐induced permeable vesicles based on poly(2‐cinnamoylethyl methacrylate) (PCEMA)‐*b*‐PNIPAM.^53^ The hydrophobic PCEMA block was crosslinked by UV light irradiation, resulting in the formation of chemically crosslinked polymer vesicles. Vesicle‐encapsulated 4‐aminopyridine (used as a model compound) was rapidly released below its LCST (25 °C), and the release rate decreased above its LCST (50 °C). This correlated with a shift from hydrophobic to hydrophilic PNIPAM chains, which caused hydration and swelling of the membrane. This phenomenon enables substrates to pass through membranes and provides systems with on–off permeability regulation. This concept has been used to develop other temperature‐responsive polymer vesicle systems.^54^ However, biocatalytic nanoreactors based on temperature‐responsive vesicles have not yet been obtained.

#### 2.2.2 Polymer Vesicles with Reconstituted Membrane Proteins

As mentioned in Section 2.2, the incorporation of channel membrane proteins into lipid vesicles is a promising approach to achieving permeability. However, block copolymer membranes are much thicker than lipid bilayer membranes. The sizes of the hydrophobic regions in membrane proteins are designed to fit the thicknesses of lipid membranes. The incorporation of membrane proteins into polymer vesicle membranes was therefore believed to be too difficult. However, Meier et al. reported incorporation of a channel protein (bacterial porin OmpF) into poly(2‐methyloxazoline)‐*b*‐poly(dimethylsiloxane)‐*b*‐(2‐methyloxazoline) triblock copolymer bilayer membranes with retaining channel activities.^55^ The polymers in the membranes are highly flexible and can adapt to the dimensions of the channel proteins.^56^ Since this discovery, other channel proteins^57^ have been incorporated into polymer vesicle membranes to enhance their permeability.^58^

In 2005, Ranquin et al. showed that polymer vesicles equipped with channel membrane proteins could be used to create biocatalytic nanoreactors.^10^ These nanoreactors were functionalized by encapsulating the purine‐specific nucleoside hydrolase of *Trypanosoma vivax* (TvNH) and permeabilizing with channel membrane proteins (OmpF, Tsx). Added prodrugs such as 2‐fluoroadenine were successfully hydrolyzed, which shows that the prodrugs diffused into the vesicle interiors through the membrane protein and were activated by TvNH.

**Figure 7:**
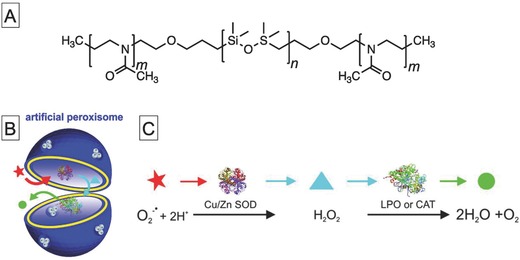
A) Chemical structure of amphiphilic polymer that can incorporate channel protein OmpF into polymer vesicle membrane. B) Schematic diagram of artificial peroxisomes that contain a set of antioxidant enzymes (SOD and lactoperoxidase (LPO) or CAT) in polymer vesicle with channel membrane proteins, and C) schematic enzymatic cascade reaction occurring inside artificial peroxisomes and serving to detoxify superoxide radicals and related H_2_O_2_. Reproduced with permission.^60^ Copyright 2013, American Chemical Society.

Hunziker and co‐workers further elaborated the system and created cell‐specific integrating biocatalytic nanoreactors.^59^ This was achieved by functionalization with the oligonucleotide poly(guanylic acid) [poly(G)] of the outer surfaces of polymer vesicles with encapsulated trypsin. The specific interactions between macrophage scavenger receptors and poly(G) enabled the vesicles to be internalized into only macrophages of a specific class; they accumulated in the endoplasmic reticulum (ER) and Golgi. To evaluate the biocatalytic activity of the nanoreactors, all regular cellular trypsin activity was inhibited by addition of specific trypsin inhibitors to the medium. Despite this treatment, the trypsin activity of the cells treated with trypsin‐loaded vesicles remained intact, which shows that the system can act as a biocatalytic nanoreactor in specific organelles, i.e., artificial organelles. Palivan and co‐workers also used this system to prepare polymer vesicles with encapsulated superoxide dismutase (SOD) and peroxisome to act as artificial peroxisomes for detoxifying superoxide radicals and H_2_O_2_ inside living cells (**Figure** **7**).^60^

For the majority of reported polymer vesicles with incorporated channel membrane proteins, low‐molecular‐weight molecules can always pass through the channel. The enzymatic reaction can therefore occur everywhere when the substrates are present. From this perspective, on‐demand enzymatic reactions inside vesicles are favored over traditional permeable nanoreactors because they enable spatiotemporal drug production, which can enhance therapeutic effects and reduce side effects. Palivan and co‐workers developed biocatalytic nanoreactors with pH‐triggered enzyme activation systems based on poly(2‐methyloxazoline)‐*b*‐poly(dimethylsiloxane)‐*b*‐poly(2‐methyloxazoline) polymer vesicles embedded with chemically modified channel porins.^61^ They introduced Cy5 dye into the amino acid residues located inside the constricted zones of the OmpF pores via an acid‐labile hydrazone linker. The chemically modified OmpF served as a “gate,” enabling pH‐driven molecular flow through the membrane.

Although functionalization of polymer vesicles with natural channel proteins is a robust method for preparing permeable vesicles, this approach requires specific amphiphilic polymers capable of incorporating membrane proteins, and the preparation of membrane proteins is complicated and is not scalable. Additionally, it is difficult to control the pore size or pore surface properties. In addition to the use of natural membrane proteins as channels for small molecules, artificial channel molecules have also recently been used to render polymer vesicles permeable. An elegant example, reported by Battaglia and co‐workers, involved the use of DNA‐based nanochannels.^62^ The DNA nanochannel consisted of six interconnected DNA duplexes and had three cholesterol groups at its outer surface, enabling insertion into polymer vesicle membranes. The channel had the outer dimensions 9 nm × 6 nm and a lumen diameter of 2 nm, allowing diffusion of small molecules. The DNA nanochannel was successfully inserted into the membranes of polymer vesicles, which were based on poly[2‐(methacryloyloxy)ethylphosphorylcholine]‐*b*‐poly(diisopropylaminoethyl methacrylate) (PMPC‐*b*‐PDPA). They proved that a nanoreactor with encapsulated trypsin can transport a fluorogenic enzyme substrate into its lumen through the DNA nanochannel, followed by hydrolysis of the substrate by trypsin. A DNA‐based nanochannel has some advantage over natural channel proteins, such as scalability and controllability of the pore size and physical properties of the pore surface (e.g., charge and hydrophilicity). With the rapid development of DNA nanotechnology, it is envisioned that DNA‐based nanochannels will attract increasing attention.

#### 2.2.3 Intrinsically Permeable Polymer Vesicles

In addition to polymer vesicles with permeability triggered by stimuli and channel protein insertion, intrinsically permeable vesicles have been reported. The first example of intrinsically permeable polymer vesicles was developed by Nolte and co‐workers in 2003.^63^ The polymer consisted of PS‐*b*‐poly[3‐(isocyano‐l‐alanylaminoethyl)thiophene] (PS–PIAT). CALB encapsulated in vesicles can transport its substrate (DiFMU octanoate) into the inner cavity, followed by substrate hydrolysis. Another enzymatic oxidation reaction in nanoreactors based on such vesicles has also been reported.^64^

These polymer vesicles were used to achieve enzymatic polymerization of a series of lactones by CALB inside the vesicles.^65^ In a similar system, cascade enzymatic reactions were achieved by using GOD and HRP encapsulated in PS–PIAT vesicles.^66^ Immediately after reporting this system, the same research group developed an enzyme cascade system in which all the enzymes were present in a single polymer vesicle.^67^ This was achieved by anchoring enzymes to the surfaces of polymer vesicles, using click chemistry,^68^ and encapsulating two enzymes inside the vesicles. Louzao and van Hest also developed antioxidant nanoreactors based on SOD and HRP encapsulated in polymer vesicles, which consisted of PS–PIAT and PEG–PS.^69^

Nallani et al. fabricated multicompartmentalized cell‐mimetic nanoreactors, which consisted of a combination of PS–PIAT vesicles as outer compartments, and poly(2‐methyloxazoline) (PMOXA)–poly(dimethylsiloxane) (PDMS)–PMOXA vesicles with inserted OmpF as inner compartments.^70^ The PS–PIAT and PMOXA–PDMS–POMOXA vesicles contained GOD and HRP, respectively. On addition of glucose and amplex red, GOD inside the PS–PIAT produced gluconolactone and H_2_O_2_, followed by diffusion of H_2_O_2_ into the PMOXA–PDMS–POMOXA vesicles. The HRP in the PMOXA–PDMS–POMOXA vesicles then catalyzed oxidation of amplex red in the presence of H_2_O_2_. This clearly indicated that the multicomponent vesicles with two entrapped enzymes acted as cell‐mimetic nanoreactors.

It is worth noting that the use of PS–PIAT‐based vesicles is not limited to simple enzymatic reactors or cell‐mimetic reactors. A study that focused on the applications of intracellular biocatalytic nanoreactors has been reported. In this study, van Hest and co‐workers fabricated PS–PIAT vesicles functionalized with cell‐penetrating trans‐activator of transcription (TAT) peptide to enhance cellular uptake.^71^ They showed that nanoreactors with encapsulated HRP were internalized into HeLa cells via macropinocytosis and that these reactors catalyzed oxidation of 3,3′,5,5′‐tetramethylbenzidine inside living cells.

**Figure 8:**
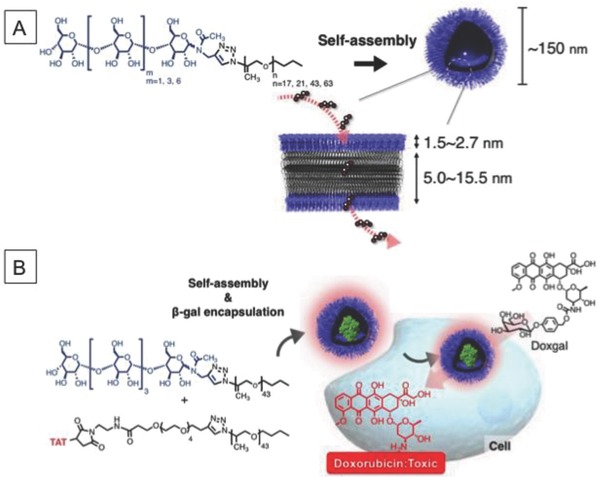
A) Chemical structure of amphiphilic maltooligosaccharide‐*b*‐poly(propylene glycol) (PPG) with schematic diagram of self‐assembly of polymers to form permeable polymer vesicles and B) schematic diagram of coassembly of maltopentaose‐containing PPG and TAT‐modified PPG, and hydrolysis of doxorubicin prodrug by β‐galactosidase‐loaded TAT–CAPsomes in living cells. Reproduced with permission.^72^ Copyright 2017, Wiley‐VCH.

More recently, we developed intrinsic permeable polymer vesicles based on carbohydrate‐*b*‐poly(propylene glycol) (**Figure** **8**).^72^ The glycopolymer self‐assembles in aqueous solution into a unilamellar vesicle (referred to as CAPsome) of average diameter 100–150 nm. The CAPsome can encapsulate proteins (e.g., β‐galactosidase, chymotrypsin, and HRP) without sacrificing their activity. In contrast to other vesicles, a CAPsome has molecular weight–dependent molecular permeability without any chemical modification. Low‐molecular‐weight compounds (less than 5 × 10^3^ g mol^−1^) diffuse into the CAPsome. We also proved that the permeability was caused by the partition of molecules into the polymer membrane. This enhanced partition can be ascribed to the weakly hydrophilic nature of poly(propylene glycol). This property enables CAPsomes to act as enzyme reactors that can supply enzyme substrates to the inner aqueous phase. Furthermore, TAT peptide–functionalized β‐galactosidase‐loaded CAPsomes were effectively internalized into HeLa cells and transformed doxorubicin and 5‐fluorouridine prodrugs, leading to cytotoxicity. Moreover, the β‐galactosidase‐loaded CAPsomes acted as nanofactories, which accumulated at tumor sites and transformed a doxorubicin‐based prodrug into doxorubicin, inhibiting tumor growth. This therapeutic application will be described in more detail in Section 3.

Recently, O'Reilly and co‐workers reported inherently permeable polymer vesicles composed of PEG‐*b*‐poly(2‐hydroxypropyl methacrylate).^73^, ^74^ The polymer vesicles were prepared by polymerization‐induced self‐assembly in aqueous solution. During this process, enzymes such as HRP and GOD were loaded into the inner layer of the vesicle. Although the thickness of the membrane is ≈25 nm, which is larger than those of other vesicle membranes, enzyme substrates can permeate through the membrane, allowing enzyme‐loaded vesicles to act as enzyme reactors.

### 2.3 Polymer Capsules

In addition to lipid and polymer vesicles, polymer capsules have also been used as compartments for biocatalytic reactors. Polymer capsules are generally made of hydrophilic polymers and have semipermeable shells. Several approaches have been devised for the fabrication of polymer capsules. In the following section, we describe three key types of polymer capsule: layer‐by‐layer (LbL)‐assembled capsules, polyion complex vesicles, and enzyme nanocapsules. A number of excellent reviews of the fabrication and application of LbL capsules, and their therapeutic cargos, have been published,^75^, ^76^, ^77^, ^78^ therefore we will discuss only the key features of LbL capsules and their use in biocatalytic nanoreactors.

#### 2.3.1 Layer‐by‐Layer‐Assembled Capsules

Among various approaches for capsule fabrication, one of the most reported methods is LbL assembly. This approach was first used by Caruso et al. in 1998.^79^ Their method relies on stepwise adsorption of polyelectrolytes onto a sacrificial template, followed by decomposition of the template. The chemical and physical properties of such LbL capsules can be easily controlled.^80^, ^81^ For example, the capsule size can be tuned in the range from nanometers to micrometers by changing the size of the sacrificial template. Their surface charges, shell thicknesses, and shapes can also be modulated. This method for LbL capsule fabrication is not limited to electrostatic interactions. Hydrogen bonding,^82^, ^83^ covalent interactions,^84^ and, more recently, chelating interactions^85^, ^86^ have also been used to fabricate LbL capsules.

The most important feature of LbL capsules is their inherent semipermeability.^87^ The capsule shell allows the passage not only of low‐molecular‐weight compounds such as dyes and ions, but also of high‐molecular‐weight compounds, including polysaccharides and proteins.^88^ More interestingly, this permeability depends on the shell thickness, porosity, and components. This indicates that the permeability can be tuned on the basis of these factors. For example, the permeability of poly(styrene sulfonate) (PSS)/poly(allylamine) (PAH) capsules by fluorescein decreased with increasing shell thickness.^89^ Moreover, an increase in the ionic strength enhanced the permeability by weakening the electrostatic interactions between polyelectrolytes in the shell.^87^

Because of their hollow structures and the semipermeability, and easy control of their chemical and physical properties, LbL capsules have been used as biocatalytic nanoreactors. Enzyme‐loaded LbL capsules are produced by three main approaches: 1) electrostatic deposition of the enzyme (enzyme incorporation), 2) enzyme preloading within a template, and 3) postloading of the enzyme inside the LbL capsules.

The first strategy is based on incorporation of enzymes into electrostatically assembled shells.^76^ Enzymes are essentially polyelectrolytes and their charge can be changed by changing the pH, therefore they can be incorporated into LbL capsules as cationic or anionic components. For example, Möhwald and co‐workers reported α‐chymotrypsin‐loaded LbL capsules.^90^ They prepared LbL capsules by stepwise absorption of PSS and PAH onto a melamine formaldehyde (MF) core, followed by degradation of the MF core with an acidic solution. The resulting LbL capsules retained negatively charged PSS/MF complexes on the capsule walls and/or inside the capsules, even after treatment. Addition of α‐chymotrypsin at an acidic pH led to successful entrapment of α‐chymotrypsin in the capsules. This is ascribed to electrostatic deposition of α‐chymotrypsin on the negatively charged PSS/MF complexes inside the capsules. Because of the superior permeability of the LbL membrane, the α‐chymotrypsin‐loaded capsule can act as a nanoreactor for the oxidation of 2,2′‐azino‐bis(3‐ethyl benzothiazoline‐6‐sulphonic acid) (ABTS) by H_2_O_2_. Importantly, ≈90% of the original catalytic activity of the loaded α‐chymotrypsin was retained even after incubation for 3 h at 47 °C. The same research group used the same method to develop HRP‐loaded LbL capsules.^91^ Although this method is applicable to a wide variety of enzymes, it has the drawback of decreased enzymatic activity because of electrostatic interactions with the shell components.

The second strategy relies on physical absorption onto templates such as mesoporous silica and CaCO_3_. These templates have porous structures, therefore enzymes can be adsorbed in the pores. For example, mesoporous silica–templated LbL capsules can be loaded with a wide variety of enzymes, including CAT and lysozyme.^92^, ^93^ These mesoporous silica–templated LbL capsules have one drawback for use as biocatalytic reactors, i.e., the difficulty of decomposing mesoporous silica. The mesoporous silica template is generally decomposed by treatment with hydrofluoric acid, which might denature the enzyme. In contrast, CaCO_3_ can easily be decomposed by treatment with ethylenediaminetetraacetic acid (EDTA).^94^ Wu and co‐workers prepared CAT‐loaded LbL capsules by using a CaCO_3_ sacrificial template.^95^ The CAT‐loaded CaCO_3_ template was prepared by mixing CaCl_2_ and Na_2_CO_3_ in the presence of CAT. LbL coating with PSS and protamine of the CAT‐loaded CaCO_3_ was then performed, followed by silica layer coating to enhance stability. Finally, the CaCO_3_ template was removed by dissolution with EDTA to yield CAT‐loaded LbL capsules. When H_2_O_2_ was added to the capsule solution, H_2_O_2_ decomposition was observed, indicating that the CAT‐loaded capsule acted as a biocatalytic microreactor. Konrad and co‐workers used a similar method to prepare asparginase (ASNase)‐loaded LbL capsules.^96^ In this case, dextran sulfate and poly‐l‐arginine were used as polyelectrolytes. They also showed that the ASNase‐loaded capsules retained their enzymatic activity and inhibited growth of leukemic cells by depletion of l‐asparginase in the medium. The limitation of CaCO_3_ templates is the difficulty in controlling its size, which is generally several micrometers.

The third strategy for enzyme encapsulation is based on the capsule permeability. In this approach, enzymes are loaded into already formed capsules by altering the shell permeability. This can be achieved by stimuli‐responsive LbL capsules that can switch their permeability from open to closed states. Capsules with permeabilities that can be reversibly switched in response to pH,^84^, ^97^ temperature,^98^ and ionic strength^98^ have been developed. As well as external stimuli, changes in solvent polarity can be used to switch the capsule permeability.^99^, ^100^ PSS/PAH‐based capsules were poured into a water/ethanol mixed solvent to form pores in the capsule wall, allowing diffusion of an enzyme (uricase) into the capsules. Subsequently, their permeability was decreased by changing the solvent to water. This enabled encapsulation of uricase inside the capsules. These strategies are applicable to encapsulation not only of enzymes but also of small molecules and other biomolecules. However, they suffer from low encapsulation efficiency.

**Figure 9:**
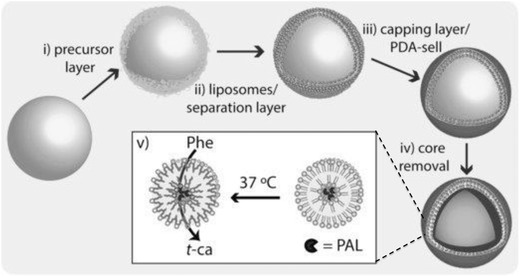
Schematic diagram of preparation and function of enzyme‐loaded microreactor. i) Layer‐by‐layer assembly of poly(l‐lysine) (PLL)/poly(methacrylic acid)‐co‐(cholesteryl methacrylate) (PMAc) polymer, using silica particle template; ii) deposition of phenylalanine ammonia lyase‐loaded liposomes, iii) surface capping by layer‐by‐layerassembly of PMAc layer and poly(dopamine) (PDA), and iv) removal of core template, and v) enzymatic conversion of phenylalanine (Phe) to nontoxic trans‐cinnamic acid (*t*‐ca) by phenylalanine ammonia lyase (PAL)‐loaded microreactors above *T*_m_ of liposomes. Reproduced with permission.^106^ Copyright 2017, Wiley‐VCH.

In addition to the above three approaches, another interesting approach for loading enzymes into LbL capsules is the use of lipid vesicles. Caruso and co‐workers used the enzyme‐encapsulating ability of lipid vesicles to prepare LbL capsules with embedded enzyme‐loaded lipid vesicles, i.e., capsosomes.^101^ The semipermeable polymer shell and the enhanced permeability of the lipid membrane at the phase‐transition temperature enabled enzyme‐loaded capsosomes to act as biocatalytic reactors for the hydrolysis of nitrocefin by β‐lactamase^102^, ^103^, ^104^ and for the reduction of glutathione disulfide by glutathione reductase.^105^ The use of enzyme‐loaded capsosomes has recently been extended to extra‐ and intracellular biocatalytic reactors. Städler and co‐workers used phenylalanine ammonia lyase (PAL)‐loaded capsosomes as extracellular microreactors for the conversion of phenylalanine to *trans*‐cinnamic acid in the presence of human intestinal epithelial HT‐29 cells, as a potential oral treatment for phenylketonuria (**Figure** **9**).^106^ Intracellular biocatalytic reactors have also been reported by the same research group. They developed tripeptide Arg‐Gly‐Asp (RGD) peptide–coated CAT‐loaded capsosomes to produce H_2_O_2_, which led to reduced cell viability.^19^ More recently, they used CAT‐loaded capsosomes as microreactors for detoxification of a planar HepG2 cell culture.^107^, ^108^ Hosta‐Rigau and co‐workers further elaborated these systems and created dual enzyme–loaded capsosomes for conducting cascade enzymatic reactions in living cells.^109^, ^110^ Recently, an example of a microreactor that uses two different enzymatic pathways has been developed by Städler and co‐workers.^111^ They encapsulated five different enzymes into capsosomes and showed that cyclical enzymatic reactions were achieved with a combination of glutamate dehydrogenase and glutathione reductase for reduction of nicotinamide adenine dinucleotide phosphate (NADP^+^) and oxidation of nicotinamide adenine dinucleoide phosphate hydrogen (NADPH), and cascade reactions were achieved with β‐galactosidase, glucose oxidase, and catalase, with lactose as a substrate. This mimics the biocatalytic ability of cells.

Although these studies clearly show that LbL‐based capsules are promising candidates for biocatalytic reactors, only a few examples of in vitro biocatalytic reactors have been reported as described above.^96^, ^106^, ^107^, ^108^, ^109^, ^110^ This might be because of stability issues with the capsules, which degrade under physiological conditions. New ideas are therefore needed for improving the stability without decreasing the semipermeability of LbL capsules.

#### 2.3.2 Polyion Complex Vesicles

Another elegant example of permeable polymeric compartments was reported by Kishimura and co‐workers.^112^, ^113^ They used polyion complexes of PEG–poly(α,β‐aspartic acid) and PEG–poly([2‐aminoalkyl‐α,β‐aspartamide] to produce polymer vesicles, referred to as PICsomes. These were easily prepared by simply mixing the polymers in aqueous solution, and they have semi‐permeable polyion complex membranes. The permeability of PIC membranes is molecular‐weight dependent and allows permeation of up to 70 kDa fluorescein isothiocyanate (FITC)–dextran. Nanoreactors based on Mb encapsulated in PICsomes can therefore be produced.^114^ The encapsulated Mb is readily reduced to deoxyMb by permeated S_2_O_4_^2−^ from the outer vesicular environment and the oxidized and reduced states of Mb can be reversibly controlled by alternating bubbling of O_2_ and Ar gas into the nanoreactor solution. Another enzymatic nanoreactor system, which is based on β‐galactosidase encapsulated in PICsomes, was also reported.^115^

The membrane permeability can be tuned by changing the pH^116^ and crosslinking of the membrane by formation of amide bonds between amine and carboxyl groups of cationic and anionic polymers, respectively.^117^, ^118^ The resulting crosslinked PICsomes have improved stability under physiological salt conditions and retain their size and distribution in a solution containing 150 × 10^−3^ m NaCl. In addition to this solution stability, the PEG surface of the PICsome enables long‐term blood circulation in vivo.^119^ It is worth noting that selective normal and tumor tissue accumulations were achieved by tuning the PICsome size. PICsomes of average diameter greater than 150 nm showed negligible tumor accumulation and significant spleen accumulation, whereas PICsomes of size less than 150 nm showed increased tumor accumulation and decreased spleen accumulation.

**Figure 10:**
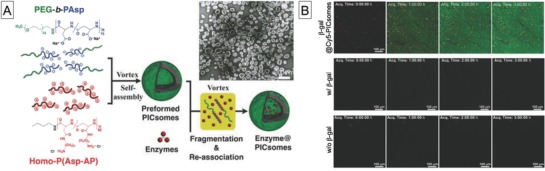
A) Chemical structures of polymers that form PICsomes and preparation of enzyme@PICsomes via preformed PICsomes, and transmission electron microscopy image of β‐galactosidase@PICsomes, and B) real‐time observation of enzymatic reaction in C26 tumor tissue by intravital real‐time confocal laser scanning microscopy. Reproduced with permission.^120^ Copyright 2016, Wiley‐VCH.

These PICsome characteristics were used to develop in vivo nanoreactors (**Figure** **10**). For example, PICsomes with encapsulated β‐galactosidase were used for conversion of a fluorogenic substrate (HMDER‐β‐galactosidase).^120^ β‐Galactosidase encapsulated in PICsomes was systemically injected into CT‐26 tumor‐bearing mice and was successfully accumulated in the tumor region. Its enzymatic activity was retained even 4 days after administration, confirmed by detection of fluorescence derived from hydroxymethyl diethylrhodol (HMDER) by in vivo imaging system (IVIS) and intravital real‐time confocal microscopy. More recently, a system with ASNase encapsulated in PICsomes was developed.^121^ Because some leukemic cells are deficient in asparagine synthetase, the removal of asparagine has an antiproliferative effect on leukemic cells. A system capable of removing l‐aspargine in the bloodstream was therefore developed. ASNase encapsulated in PICsomes retained its enzymatic activity and achieved enzymatic hydrolysis of aspargine after systemic injection into mice, because of long‐term circulation in the blood.

#### 2.3.3 Enzyme Nanocapsules

The strategies for enzyme encapsulation described above are generally based on entrapment of enzymes in already formed compartments such as vesicles. Although this approach can be used for facile preparation of enzyme‐loaded compartments, it suffers from low encapsulation efficiency. This problem, which is related to passive encapsulation of enzymes during compartment formation, was solved by Lu and co‐workers.^122^ Enhanced encapsulation efficiency was achieved by polymer shell formation on the enzyme surface. Such enzyme‐templated nanocapsules were prepared by introduction of acrylate groups into enzymes, followed by radical polymerization of the surface of the acrylated enzymes in the presence of 2‐dimethylaminoethyl methacrylate, acrylamide, and *N*,*N′*‐methylene bisacrylamide. The surface charge on the capsules can be adjusted by varying the ratio of the cationic and neutral monomers. Degradability can also be incorporated by introducing glycerol dimethacrylate. This is a facile procedure, and the authors showed that a wide variety of enzymes, including enhanced green fluorescent protein, HRP, bovine serum albumin (BSA), SOD, and caspase‐3 could be used as enzyme templates. Enzyme nanocapsules were effectively internalized into cells and escaped from endosomes, possibly because of the proton sponge effect. Furthermore, the enzyme nanocapsules retained their enzymatic activity inside living cells and even in the tissues of capsule‐injected mice. The authors have elaborated this system to develop reduction‐123 and enzyme‐responsive^124^ enzyme nanocapsules and have used them for organophosphate removal.^125^ They also showed the utility of this system as a therapeutic biocatalytic reactor for alcohol intoxication.^126^ This will be described in more detail in Section 3.

### 2.4 Mesoporous Silica Particles

Mesoporous silica particles (MSPs) provide a solid framework with a porous structure. MSPs are generally prepared by templating methods, using surfactants and degradable solid materials. They can be tailored to give various structures and morphologies by controlling the reaction conditions such as temperature, pH, template, and silica source.^127^ MSPs have pores of diameter 2–50 nm, which are suitable for enzyme entrapment and substrate permeation.^128^, ^129^ MSPs are therefore attractive materials for biocatalytic reactor compartments. Several studies have focused on the development of MSP‐based biocatalytic nanoreactors, which can encapsulate or immobilize enzymes and convert enzyme substrates.

**Figure 11:**
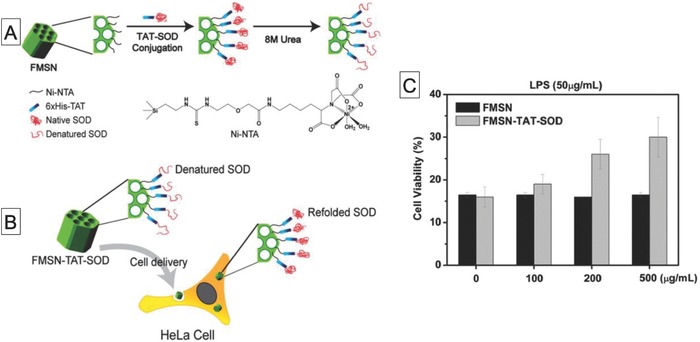
A) Schematic diagram of preparation of Ni–NTA‐modified mesoporous silica nanoparticles (FMSNs) with surface functionalization by histidine‐tagged TAT–SOD protein, B) refolding of denatured SOD on FMSNs after cell internalization, and C) viabilities of HeLa cells treated with lipopolysaccharide, a ROS inducer, in the presence of FMSN–TAT–SOD or FMSNs. Reproduced with permission.^131^ Copyright 2013, American Chemical Society.

Trewyn and co‐workers reported Au nanoparticle–capped MSPs for intracellular delivery of an enzyme and a substrate.^130^ They used channel‐like mesoporous nanoparticles as a container for luciferin and luciferase. Luciferin was loaded into the pores and was confined by capping disulfide‐linked Au nanoparticles, and luciferase was electrostatically adsorbed on the MSP surfaces. These MSPs with a coencapsulated enzyme and substrate were successfully internalized into living cells, as confirmed by confocal laser scanning microscopy and the codelivered luciferase was converted to luminescent oxyluciferin in vitro. The enzyme‐loaded MSPs were internalized into living cells, but the uptake efficiency was relatively low. To tackle this problem, Mou and co‐workers fabricated TAT peptide–functionalized MSPs for enhanced intracellular delivery of enzymes (**Figure** **11**).^131^ Their strategy relies on functionalization of Ni–nitrilotriacetic acid (NTA) on mesoporous silica particles, followed by immobilization of a histidine‐tagged TAT–SOD hybrid protein. As expected, the delivery efficiency of SOD–TAT‐functionalized MSPs was much better than that of control MSPs. When HeLa cells were exposed to a lipopolysaccharide, i.e., a ROS inducer, the cell viability increased and the activation of cleaved caspase‐9 and p21 proteins decreased, suggesting that SOD–TAT‐functionalized MSPs reduced ROS concentrations in vitro. In the follow up work, an additional enzyme, glutathione peroxidase, was used to enhance ROS scavenging.^132^ Although these studies clearly show that silica nanoparticles are promising tools for delivering enzymes into living cells, the enzymes are immobilized on the MSP surfaces. They are therefore not protected from proteolytic degradation by endogenous enzymes, and this could cause immunogenicity. Protection of enzymes is important because it permits sustained enzymatic reactions and therefore increases the therapeutic efficiency. To this end, silica hollow nanoparticles are also used for the compartments of biocatalytic nanoreactors. For example, the same research group encapsulated HRP in silica hollow particles by a water‐in‐oil microemulsion templating approach.^133^ Because template removal was unnecessary and mild washing conditions were used, the encapsulated HRP retained high enzymatic activity inside the MSPs. MSP‐encapsulated HRP was used as an intracellular nanoreactor in a prodrug–enzyme system to convert nontoxic indole‐3‐acetic acid to toxic free radicals. A similar strategy was used for intracellular detoxification of ROS by CAT and SOD coencapsulated in MSPs.^134^ In this study, PEI‐modified CAT and SOD were encapsulated inside the hollow silica nanospheres (HSNs). In the presence of paraquat, which is a ROS inducer, enzymes encapsulated in MSPs decreased the number of dead cells by 60% by active removal of ROS. Many MSP‐based biocatalytic nanoreactors have been developed in the past few years. Various structures, including nanochannel and hollow particles, have been designed and fabricated. These studies clearly show the utility of MSPs as compartments in biocatalytic reactors, but their biocompatibility and degradability in vitro, and especially in vivo, are unclear. Accordingly, future research should include evaluation of the safety of MSPs in vivo.

### 2.5 Viral Capsids

**Figure 12:**
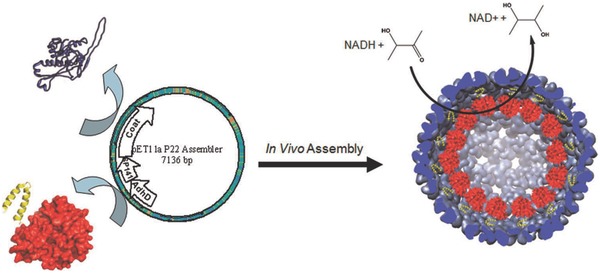
Schematic diagram of in vivo recombinant expression and encapsulation of alcohol dehydrogenase fusion inside assembled P22 capsid and reaction catalyzed by encapsulated enzyme. Reproduced with permission.^140^ Copyright 2012, American Chemical Society.

Viruses have 3D cages, called capsids, with an internal cavity that can hold genetic materials such as RNA and DNA. Viral capsids provide robust and highly monodispersed compartments with a wide variety of shapes and sizes ranging from several nanometers to micrometers. Moreover, they can encapsulate a broad spectrum of compounds, including DNA, RNA, inorganic materials, and proteins. Viral capsids have therefore attracted interest for use in the preparation of nanoreactors.^135^ In 2007, van Hest and co‐workers reported the first viral capsid–based biocatalytic nanoreactor.^136^ This was achieved by the self‐assembly of potato virus X capsids with anchored CALB. Since then, many examples of viral capsid–based biocatalytic nanoreactors have been reported. For example, Cornelissen and co‐workers encapsulated HRP in capsids derived from cowpea chlorotic battle virus.^137^ The capsids have pores of size a few nanometers, therefore the fluorogenic substrate dihydrorhodamine 6G diffused inside the capsids, and hydrolysis by HRP produced a fluorescent molecule. More interestingly, the pore size increased with increasing solution pH to 7. This enables enhancement of the capsid permeability. Douglas and co‐workers used a viral system derived from *Salmonella typhimurium* bacteriophage P22, which was assembled from 420 copies of the coat protein into a capsid with the assistance of 100–330 copies of a scaffold protein.^138^ They loaded proteins (e.g., enhanced green fluorescent protein (EGFP), mCherry) into the capsids by a genetically based system, which relied on fusion of cargo proteins with P22 proteins.^139^ They used this strategy to prepare alcohol dehydrogenase encapsulated in P22 capsids for 3‐hydroxy‐2‐butanone (acetoin) reduction^140^ (**Figure** **12**), and nicotinamide adenine dinucleotide (NADH) oxidase encapsulated in P22 capsids to produce H_2_O_2_ for bacterial growth inhibition.^141^ Recently, Vazquez‐Duhalt and co‐workers reported prodrug transformation by a biocatalytic virus inside living cells.^142^ They also used a genetically fused system to produce a fusion protein containing cytochrome P450 (CyP) and a viral scaffold protein from bacteriophage P22. These proteins were successfully coassembled in enzyme‐encapsulating virus‐like compartments. These compartments were further functionalized with PEG–folate to give cell specificity. The folate functionality enabled the CyP‐loaded capsids to convert 7‐benzyloxy‐4‐trifluoromethylcoumarin to fluorescent 7‐hydroxy‐4‐trifluoromethylcoumarin in HeLa and MCF‐7 cells. Moreover, activation of a tamoxifen prodrug in living cells induced significant cytotoxicity.

Although viruses and their capsids have been widely used in nanomedicine because of their good transporting abilities,^143^ monodispersed size and shape, and encapsulation abilities, their use is hindered by potential safety issues such as pathogenicity and immunogenicity. These issues need to be addressed in future studies to enable expansion of the use of viruses and their capsids.

### 2.6 Hydrogels and Polymer Films

**Figure 13:**
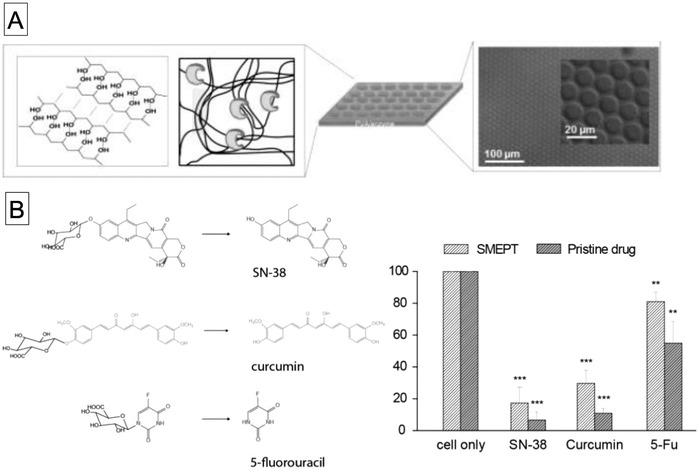
A) Schematic diagram of structure of enzyme‐functionalized physical hydrogels based on poly(vinyl alcohol) and differential interference contrast image of hydrogels and B) chemical structures of glucuronide‐modified prodrug and native drug, and viabilities of hepatocyte cells cultured in presence of prodrugs and β‐Glu‐loaded hydrogels. Reproduced with permission.^146^ Copyright 2014, Wiley‐VCH.

Hydrogels, which are 3D networks of crosslinked polymer chains, have been widely investigated as promising scaffolds in regenerative medicine because of their high biocompatibility, similarities to the native extracellular matrix, and their abilities to encapsulate a wide variety of molecules, including peptides and proteins.^144^, ^145^ Low‐molecular‐weight compounds can penetrate inside a hydrogel because of the network structure, therefore hydrogels can entrap enzymes and are ideal for use as biocatalytic reactor scaffolds. The advantage of such hydrogel‐based biocatalytic nanoreactors over typical particle‐based reactors is that the reactors can be physically placed on a desired site such as tumor tissue. Particle‐based biocatalytic reactors are generally directed to the disease site by systemic injection. This method is successful and can be adapted to deliver reactors to metastasized tumor tissues, but only a few nanoreactors can reach the desired site. This decreases their therapeutic efficacy. In contrast, hydrogels are implantable materials that can deliver large amounts of enzymes locally, which enhances the therapeutic effect. In a seminal study, Mendes and Zelikin developed biocatalytic hydrogels based on poly(vinyl alcohol) (PVA) with embedded β‐Glu for substrate‐mediated DDSs (**Figure** **13**).^146^ They showed that hydrogels with entrapped β‐Glu could convert three different anticancer prodrugs to cytotoxic drugs in HepG2 cell culture media, leading to an antiproliferative effect. They also reported that the biocatalytic hydrogels transformed two different prodrugs. They used this to achieve two different anti‐inflammatory and antiproliferative effects at a desired time by sequential addition of curcumin–Glu and dexamethasone–Glu to a cell culture medium containing the biocatalytic hydrogel. Moreover, simultaneous coadministration of curcumin–Glu and SN‐38–Glu enhanced their therapeutic effects. Recently, Zelikin and co‐workers developed EPT‐based systems consisting of two enzymes encapsulated in calcium alginate hydrogel beads for drug elution.^147^ Alginate hydrogel beads equipped with β‐galactosidase and β‐glucuronidase were successfully used to synthesize both NO and an anticancer drug (SN‐38), and therefore gave vasodilation and antiproliferation.

Hydrogels are a superior platform for embedding not only synthetic vesicles (liposomes) but also naturally occurring vesicles, i.e., extracellular vesicles (EVs) or exosomes. Stevens and co‐workers recently reported that EVs with encapsulated β‐glucuronidase were successfully embedded in a PVA‐based hydrogel without sacrificing the enzymatic activity and showed that the EV–hydrogel hybrid produced anti‐inflammatory drugs (curcumin) upon addition of curcumin‐β‐d‐glucuronide.^148^ Exosomes are involved in numerous physiological functions, therefore this exosome‐localizing technique is a promising approach for exosome‐based therapeutics.

At around the same time, the same research group showed that this strategy could be applied to polymer films produced by LbL assembly.^149^, ^150^ The biocatalytic films were prepared by sequential deposition of five poly(l‐lysine) (PLL)/poly(methacrylic acid) (PMA) layers, followed by incubation of β‐Glu and subsequent adsorption of another four PLL/PMA layers. The biocatalytic activity of the film was evaluated on the basis of activation of SN‐38–Glu in a HepG2 cell culture medium. The cell viability decreased to 80% when the cells were cultured on the biocatalytic film in the presence of SN‐38–Glu, indicating that the prodrug was successfully converted to an anticancer drug. The biocatalytic film was able to transform the prodrug under flow conditions. This enabled site‐specific prodrug activation with cells cultured in sequentially connected flow chambers. They have recently taken biocatalytic films to the next level by using surface coating of metallic wires in combination with their film‐based EPT technique.^151^ Nitric oxide (NO) regulates a wide variety of physiological activities such as inflammation, vasodilation, and antiproliferation. Although NO has potential as a therapeutic agent, its lifetime is very short (≈1 s) in human tissues and biological environment. It is therefore necessary to generate NO near the disease site. They therefore coated metal wires with biocatalytic films with embedded β‐galactosidase. This enables delivery of the film at target sites through blood vessels. Such biocatalytic metal wires enabled localized enzymatic transformation of β‐gal–diazeniumdiolate (NONOate) to produce NO molecules. They also achieved ex vivo contraction of rat artery with their biocatalytic wires.

## 3 In Vivo Therapeutic Biocatalytic Nanoreactors

As discussed in Section 2, there are a large number of reports of the development of biocatalytic nanoreactors based on lipid and polymer vesicles, capsules, mesoporous silica, viral capsids, and hydrogels. However, only a few studies of biocatalytic reactors for in vivo therapeutic applications have been reported. In the following sections, recent advances in the development of biocatalytic reactor–based therapies for cancer, glaucoma, neuropathic pain, and alcohol intoxication will be discussed.

### 3.1 Cancer

Cancer chemotherapies based on biocatalytic nanoreactors offer advantages over conventional DDSs. Biocatalytic nanoreactors can transform prodrugs into the original drugs in situ, which can maximize the therapeutic efficacy and minimize side effects.

For example, we recently reported an enzyme prodrug activation system for cancer therapy, which is based on β‐galactosidase‐loaded polymer vesicles with inherent permeable membranes, as described in Section 2.2.3.^72^ The polymer vesicles (CAPsomes) showed molecular weight–dependent permeability, enabling enzyme encapsulation and permeation of low‐molecular‐weight substrates. For therapeutic purposes, we used a combination of β‐galactoside‐modified doxorubicin as a prodrug and β‐galactosidase‐loaded CAPsomes as a biocatalytic nanoreactor. The prodrug activation efficacy of this system was confirmed in vitro with various cancer cell lines, including HeLa, CMS5a, and CT26, and in vivo with a CT26‐bearing mouse model, in which tumor inhibition was confirmed by a significant reduction in tumor volume. More importantly, no significant systemic toxic effects (i.e., weight loss, acute organ toxicities) were observed.

**Figure 14:**
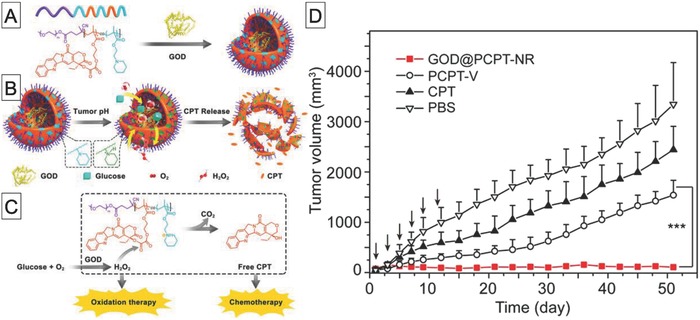
A) Chemical structure of amphiphilic polymer containing camptothecin and pH‐responsive segments, B) schematic diagram of preparation of GOD encapsulated in polymer vesicles, C) molecular mechanism of release of H_2_O_2_ and camptothecin, and D) tumor growth profiles of A549 tumor‐bearing mice treated with PBS, free camptothecin, polymer vesicles, and GOD‐loaded polymer vesicles. Reproduced with permission.^153^ Copyright 2017, American Chemical Society.

Although our system significantly inhibited tumor growth, the inhibition efficacy was relatively low. The main reason could be different biodistributions of the prodrug and nanoreactors. Additionally, it has been reported that for cancer monotherapy, it is difficult to eliminate the whole tumor and to prevent cancer metastasis.^152^ To overcome this drawback of monotherapy, combined therapies, which involve combinations of two or more therapies, have been developed. The use of biocatalytic reactors for combined cancer therapy has been reported. For example, Ge and co‐workers developed prodrug‐based polymer vesicles loaded with GOD, which can deliver a combination of chemotherapy and oxidation therapy for cancer (**Figure** **14**).^153^ Their polymer was PEG‐*b*‐poly[(2‐methacryloyloxy)ethylcamptothecin oxalate]‐*co*‐poly[2‐(piperidin‐1‐yl)ethyl methacrylate) [PEG‐*b*‐poly(CPTMA‐*co*‐PEMA)]. Poly(PEMA) serves as a pH‐responsive unit and can switch from hydrophobic to hydrophilic with pH changing from 7.4 to 6.8. This change enables permeation of low‐molecular‐weight compounds. The poly(CPTMA) segment was designed to release camptothecin, which is an anticancer drug, on treatment with H_2_O_2_. Polymer vesicles with encapsulated GOD accumulated at the tumor site, because of the enhanced permeation and retention (EPR) effect, and produced H_2_O_2_ from glucose by the enzymatic reaction with GOD in situ. The resulting H_2_O_2_ hydrolyzed the linker between the polymer and camptothecin, leading to camptothecin release. The produced H_2_O_2_ and camptothecin at the tumor site both significantly inhibited tumor growth. Furthermore, H_2_O_2_ production involves consumption of glucose, which is a major nutrient for cancer, and therefore induces starvation. This could also enhance the antitumor effect. At almost the same time, the same researchers developed another type of biocatalytic nanoreactor for combined cancer therapy.^154^ They replaced the poly(CPTMA) segment in the PEG‐*b*‐poly(CPTMA‐*co*‐PEMA) block copolymer with a poly(phenyl boronic acid ester) [poly(PBEM)] segment. The poly(PBEM) segment was degraded by exposure to H_2_O_2_, accompanied by release of quinone methide (QM). QM can deplete intracellular glutathione, and this weakens the antioxidative properties of cancer cells. After intravenous injection of GOD‐loaded polymer vesicles, the H_2_O_2_ levels in the tumor tissue were considerably higher, and the glutathione levels were significantly lower, than those after treatment with phosphate buffered saline (PBS). This confirmed the ability of the reactor to produce H_2_O_2_ and quinone methide. This synergistic effect resulted in thorough suppression of A549 tumor growth.

The scaffold for a cancer therapeutic reactor is not limited to polymer vesicles. For example, Shi and co‐workers used dendritic mesoporous nanoparticles (DMSNs) as a reactor scaffold.^155^ Because of their highly porous nature, DMSNs can be loaded with both GOD and Fe_3_O_4_. The therapeutic mechanism of such catalytic nanoreactors is based on starvation, i.e., depletion of nutrients, and generation of hydroxyl radicals. GOD oxidizes glucose in the tumor region to gluconic acid and H_2_O_2_. The resulting H_2_O_2_ reacts with Fe_3_O_4_ at acidic pH via Fenton‐like reactions to produce toxic hydroxyl radicals. This enables therapeutic nanoreactors to act in a tumor‐specific manner because of the acidic environment around the tumor. Systemic injection of DMSNs loaded with GOD and Fe_3_O_4_ significantly inhibited 4T1 and U87 tumor growth in mice. This therapeutic effect is attributed to the contribution of a cascade catalytic reaction in the tumor tissue.

Another example of a scaffold for cancer therapeutic nanoreactors is hollow mesoporous organosilica nanoparticles (HMONs).^156^ Chen and co‐workers used HMONs for coencapsulation of GOD and l‐arginine to achieve cancer starvation and gas therapy. In their strategy, GOD oxidizes intratumoral glucose to H_2_O_2_, which reacts with l‐arginine to generate NO, which is highly toxic. In vivo experimental results showed that systemic injection of HMONs with encapsulated GOD and l‐arginine into mice bearing U87MG tumors resulted in much larger tumor volume suppression than in the cases of GOD‐loaded HMONs and l‐arginine‐loaded HMONs, indicating a synergistic therapeutic effect that is better than that of H_2_O_2_ oxidation monotherapy.

### 3.2 Glaucoma

**Figure 15:**
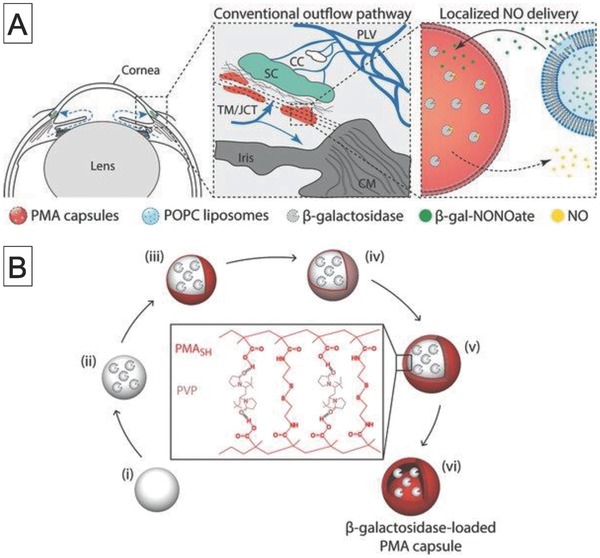
A) Schematic diagram of localized delivery of NO to conventional outflow pathway by liposomes containing NO donor (β‐galactoside–NONOate) and β‐galactosidase‐loaded PMA capsules and B) schematic diagram of preparation of β‐galactosidase‐loaded poly(methacrylic acid) capsules formed by layer‐by‐layer assembly. Reproduced with permission.^159^ Copyright 2017, Wiley‐VCH.

Glaucoma is an optic neuropathy that leads to vision loss.^157^ It has been estimated that glaucoma will affect more than 80 million individuals worldwide by 2020.^158^ The mechanism of its progression is not well understood, and intraocular pressure (IOP) is the only proven risk factor. Consequently, reducing IOP will arrest the progress of glaucoma. Use of NO, which is a signaling molecule, has been successful in lowering IOP, but its therapeutic effect strongly depends on the location and concentration of NO at the disease site. Stevens and co‐workers developed a NO delivery platform that can target the conventional outflow pathway (i.e., trabecular meshwork) and produce a controlled dose of NO (**Figure** **15**).^159^ Their strategy involves two steps: the delivery of β‐galactosidase‐loaded LbL capsules to the trabecular meshwork by intracameral injection, followed by administration of liposomes loaded with β‐galactoside–NONOate, which is a NO donor prodrug. On degradation of the liposomes, β‐galactoside–NONOate is released and diffuses into the LbL capsules, which results in NO generation. This enables dose‐dependent production of NO and increases the half‐life of NO release. A system that made use of these effects significantly improved outflow facility in enucleated mice eyes compared with that that in the case of treatment with empty capsules. This suggests that biocatalytic reactors could be used as glaucoma therapeutic materials.

### 3.3 Neuropathic Pain

Another interesting therapeutic application is neuropathic pain treatment with SOD‐loaded porous polymer vesicles,^160^ which was developed by Cheng and co‐workers.^161^ Neuropathic pain is generally caused by injury to the spinal nerve root. A recent study showed that neuropathic pain is related to damage to neural tissue by ROS.^162^ Elimination of ROS may therefore lead to a novel therapeutic approach to neuropathic pain. This goal was achieved by loading SOD, which is an antioxidant enzyme, in porous polymer vesicles based on PEG‐*b*‐poly(butadiene) and PEG–poly(propylene glycol)–PEG. The polymer vesicles are permeable, therefore ROS can diffuse into the interiors of the polymer vesicles, where they are eliminated by interactions with SOD. The withdrawal threshold of an ipsilateral injured forepaw after nerve root compression was much higher following local administration of SOD‐loaded polymer vesicles than in the case of free SOD administration. This suggests that the treatment can prevent neuropathic pain.

### 3.4 Alcohol Intoxication

**Figure 16:**
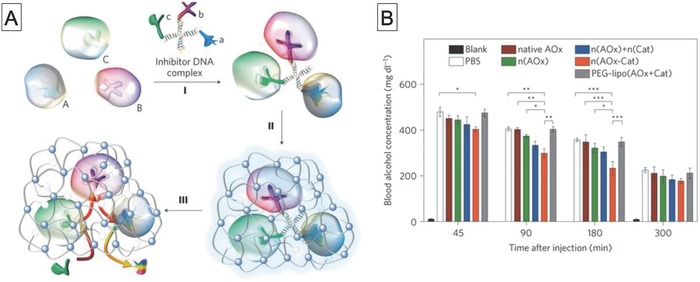
A) Schematic diagram of preparation of triple‐enzyme nanocapsule by DNA‐directed assembly, followed by in situ polymerization on surface of enzyme nanocapsules and B) blood alcohol concentrations of ethanol‐intoxicated mice treated with PBS, native alcohol oxidase (AOx), AOx nanocapsules, AOx and CAT nanocapsules, nanocapsules with coencapsulated AOx and CAT, and liposomes with coencapsulated AOx and CAT. Reproduced with permission.^126^ Copyright 2013, Springer Nature.

In addition to reactors with single encapsulated enzymes, dual enzyme–based nanoreactors for therapeutic application have also been reported. Lu and co‐workers used enzyme nanocapsules, described in Section 2.3.2. They prepared alcohol oxidase (AOx)‐ and CAT‐loaded nanocapsules by DNA‐directed assembly, followed by in situ polymerization on the enzyme surfaces, and demonstrated their therapeutic action as an alcohol antidote (**Figure** **16**).^126^ Intravenous injection into alcohol‐intoxicated mice significantly decreased their blood alcohol concentrations (BACs) compared with those of control groups, including groups treated with AOx nanocapsules, CAT nanocapsules, AOx‐ and CAT‐loaded liposomes, and native AOx. The BAC reduction was higher than that for mice treated with a mixture of AOx and CAT nanocapsules. This indicates that the enzyme nanocapsule structure, in which the enzymes are in close proximity to each other, is important. The authors concluded that this is probably because of efficient removal of H_2_O_2_, which might denature the enzymes.

## 4 Conclusions and Outlook

Therapeutic nanofactories, which are DDSs that enable therapeutic enzymatic reactions at disease sites, are becoming recognized as an important tool for biomedical applications. The aim of this review was to describe progress in the area of biocatalytic reactors and their therapeutic applications. Different kinds of compartments such as lipid and polymer vesicles, polymer capsules, mesoporous silica particles, viral capsids, and hydrogels have been used as scaffolds for biocatalytic reactors. Significant achievements from the 1980s to the present have been highlighted to introduce these concepts and inspire others to use compartments as biocatalytic reactors.

A characteristic of nanofactories is that they can be applied to a wide variety of diseases by changing the combination of an enzyme and its corresponding substrate. As discussed in Section 3, nanofactory‐based therapeutics have great potential for treating many conditions, including cancer, glaucoma, and alcohol intoxication. The rapidly increasing number of papers published in this area clearly shows the potential of nanofactory use in nanomedicine. However, the use of nanofactories as therapeutic agents is far from being a mature field. Many challenges need to be addressed to enable therapeutic nanofactories to be used in vivo, especially in preclinical and clinical trials.

So far, research has focused on the development of compartments and investigation of their applicability as enzymatic reactors in aqueous solutions or intracellular environments. A high encapsulation ability without loss of enzyme activity is important for in vitro and in vivo nanofactories. The conventional method for protein encapsulation inside lipid and polymer vesicles is film hydration. A lipid or polymer film is hydrated with a protein solution, which results in the formation of vesicles with encapsulated protein. Although this method is facile, the encapsulation efficiency is low (generally less than 10%)^163^, ^164^, ^165^ and the size of the resultant vesicles is heterogeneous. Another method relies on interactions such as electrostatic interactions between the components of the nanofactory and enzymes. In this case, high encapsulation efficiency is achieved by simply mixing a solution of charged molecules with an enzyme solution.^166^ However, this method can cause denaturation of the enzymes. A more recent method, which was reported by Battaglia and co‐workers, is based on electroporation.^167^ They used electroporation to form transient holes in polymer vesicle membranes to enable external proteins to enter the vesicles. This method achieved high encapsulation efficiencies, notably 17% for BSA and 9% for immunoglobulin G (IgG), without sacrificing protein activity. However, this method is only effective for anionic proteins. There is no versatile method for encapsulation of enzymes in compartments and suitable methods for encapsulation in individual compartments need to be developed.

In addition to the encapsulation efficiency, purification methods for compartments with encapsulated enzymes are also important because free enzymes can potentially cause side effects such as immune responses. Three purification methods are commonly used to remove free enzymes: dialysis by ultrafiltration, centrifugation, and size‐exclusion chromatography (SEC). Dialysis is based on the difference between the diffusion rates of enzymes and enzyme‐encapsulating compartments when passing through the pores in dialysis or ultrafiltration membranes. This method is simple and cheap. However, the self‐assembled compartments may decompose during this process because of their dynamic equilibrium between unimers and assemblies, which can result in a decrease in sample concentration. Centrifugation is also a facile separation technique and is based on density differences. However, centrifugation can cause aggregation of compartments. Currently, the most robust separation method is SEC, which separates enzymes and enzyme‐encapsulating compartments based on their hydrodynamic volumes. SEC columns are typically packed with sepharose or sephacryl resin and equipped with a UV–vis or refractive index detector. SEC can achieve separation and provide information on the amount of encapsulated enzyme. SEC is a reliable technique for accurately separating mixtures and is an increasingly popular technique.

Another concern for the construction of nanofactory is that stability in physiological environments also needs to be taken into account in design strategies to ensure that therapeutic enzymes are not released before delivery to the disease site. The introduction of chemical crosslinking, hydrogen bonding, or hydrophobic interactions into compartments may provide solutions for this issue. Although the construction of stabilized nanofactories is essential, it is also important for the nanofactory compartments to self‐destruct when they become unnecessary or when controlled termination of treatment is necessary. Self‐destruction facilitates elimination of nanofactory components, including encapsulated enzymes from a living body. One potential approach is to use external‐stimuli responsiveness to tune the compartment stability. Application of an external trigger such as light or heat can change the properties (e.g., polarity and structure) of stimuli‐responsive groups. This results in destruction of the compartments. Substantial progress has been made in this area, which is not intended for nanofactory construction. Detailed reviews of controlled destruction by external stimuli are available in the literature.^168^, ^169^, ^170^

Considering the benefits of systemic injection, long‐term blood circulation is another important factor. This can be achieved by surface functionalization of the compartments with biocompatible polymers such as PEG^171^, ^172^ and poly(amino acids) (e.g., polyoxazolines^173^ and polysarcosines^174^, ^175^) to reduce elimination by the mononuclear phagocytic system. Another issue is the targeting ability. As discussed in Section 3, all nanofactory cancer therapies rely on EPR for accumulation in tumors. Such passive targeting in nanomedicine is successful in small animal models, but its clinical therapeutic efficacy is limited because of the heterogeneity of cancer.^176^, ^177^ As an alternative to passive targeting based on EPR, active targeting strategies that rely on the use of specific ligands (antibodies, peptides, and saccharides) would be advantageous in enhancing accumulation in tumors, which would increase the therapeutic efficacy. The functionalization of targeting modules, which is a requirement for a nanofactory, has the potential to increase therapeutic versatility.

Recently, the delivery of enzyme‐mimetic materials such as metal catalysts and artificial enzymes (nanozymes) has attracted attention, not only for chemical labeling of living cells and tissues in living bodies,^178^, ^179^, ^180^ but also for activation of therapeutics.^181^, ^182^, ^183^, ^184^ Such nanozymes generally consist of scaffolds made of metals, or organic or organic/inorganic hybrid materials with embedded metal catalysts. They are stable and can be produced on a large scale. Additionally, fine‐tuning of the chemical structures of nanozymes enables catalysis of a wide variety of reactions, therefore nanozymes are promising and valuable materials. However, their encapsulation in stable scaffolds would make them more effective for in vivo applications. The manipulation of metal catalysts encapsulated in permeable compartments, as described in this review, will therefore be an important future direction for therapeutic nanofactories. Furthermore, combinations of natural enzymes and metal catalyst might enable the development of highly effective and safe therapeutics for clinical applications.

Overall, we have no doubt that research in the field of biocatalytic reactors, which has matured dramatically in the last 5 years, will continue to focus on the development of in vivo therapeutic nanofactories in the next few years. This new class of therapeutics holds great promise for the treatment of various diseases, and their applicability not only in therapeutics but also for sensing and diagnosis will expand. It is expected that a range of scientific communities, from chemists to medical researchers, will be involved in tackling the many challenges in this field and will enable the development of new preclinical and clinical applications in the near future.

## Conflict of Interest

The authors declare no conflict of interest.
